# The passivity breakdown of zinc antimony alloy as an anode in the alkaline batteries

**DOI:** 10.1038/s41598-022-23741-5

**Published:** 2022-11-07

**Authors:** Abd El-Rahman El-Sayed, Hoda A. El-Shafy Shilkamy, Mahmoud Elrouby

**Affiliations:** 1 Department of Chemistry, Faculty of Science, Sohag University, Sohag, 82524 Egypt; 2 Faculty of Science, King Salman International University, Ras Sudr, 46612 Sinai Egypt

**Keywords:** Chemistry, Engineering, Materials science

## Abstract

Zn is utilized as an anode in alkaline batteries because of its propensity to produce a passive colloidal layer on its surface. Then the surface should be reactivated in the passive region. Therefore, the passive state on the surface can be significantly hindered by utilizing a tiny percentage of Sb alloyed with Zn. Accordingly, the effect of minor Sb alloying with Zn on the performance of anodic dissolution and passivation in concentrated alkaline media (6 M KOH, which is used in the batteries) was studied using potentiodynamic and potentiostatic techniques. Besides, the passive layers formed at various anodic potentials were characterized utilizing scanning electron microscopy (SEM) and X-ray diffraction (XRD). The data of potentiodynamic measurements exhibited the active–passive transition curve of all studied specimens. All obtained results revealed that passivation is gradually hindered with increasing Sb content in the alloy, and less passivity was obtained at 1% Sb. Along this, a dramatic rise in current density at a particular positive potential (+ 2.0 V vs. SCE) to markedly higher values only of the electrodes containing Sb is observed.

## Introduction

In neutral media as well as alkaline batteries, zinc was successfully used as anode electrodes. The data in the literature survey^1–9^ showed that zinc-manganese, zinc-silver oxide, zinc-air, and zinc-nickel were utilized in the alkaline batteries owing to low cost, high energy, abundance, high capacity, and low toxicity. It is well known that the discharge efficiency and utilization of zinc metal as an electrode for zinc batteries are highly decreased, owing to the production of passive coating on the zinc surface and great corrosion in the concentrated alkaline solution^10–12^. In addition, the passive film formation on the surface of zinc occurs easily in the alkaline media^13^, and this colloidal film consists of ZnO and Zn(OH)_2,_ or a black film is formed on the surface, owing to dehydration of discharge product to ZnO only^14^. Zinc oxide plays an important role in electrode passivation, and the morphological variations of ZnO precipitates on the surface of the Zn anode were examined^15^. The inhibition of passivated film produced on the surface of Zn can be improved the discharge performance and the capacitance^16^. Therefore, organic and inorganic compounds^17,18^ as electrolyte or electrode additives were tested. As a result of the addition of some heavy metals (such as Cd, In, Sn, Bi, Pb, and Ti) to zinc, the hydrogen over-potential is high and leads to reduce Zn corrosion^19^. On the other hand, the dendrite growth and shape change of Zn metal were reduced by Bi alloying with Zn metal. Consequently, the capacity of Zn metal as an electrode for batteries has been improved^20,21^. Durmus et al.^22^ exhibited that 10% Al alloying with Zn leads to more corrosion resistance than that of pure Zn metal. Besides, adding different amounts of Al and Si (1–3%) to pure zinc leads to restraining the corrosion to maximum value^23^. Aremu et al.^24^ evaluated various additives simultaneously in certain tests. They showed that potassium sulfide (K_2_S) and lead oxide (PbO) could be added to Zn-BiO anodes. The measurements exhibited that the presence of BiO, PbO, and K_2_S has the ability to decrease the rate of corrosion. Also, the efficiency of the Zn-BiO-PbO-K_2_S electrode was improved. Besides, the inclusion of antimony metal in the Zn-Al alloy enhanced the corrosion resistance. This improvement in the capacity was ascribed to the higher hydrogen overpotential of antimony^25^**.** In addition, mixing carbon nitride with a Zn-Al-covered double oxide anode increased corrosion resistance^26^. Consequently, good corrosion protection can be obtained by alloying Zn with other elements^27,28^.

Antimony alloying with zinc was used in many applications; materials for Li-ion and Na-ion rechargeable batteries' anodes were fabricated from zinc-antimony alloy^29–31^. The thermoelectric characteristics of Zn-Sb thin films produced using single-element composites were investigated by Liu et al.^32^. They showed that thermal energy could be converted to electrical energy, owing to their good properties^33^. Importantly, the generation of the stabilized Zn_4_Sb_3_ phase in the alloy has an appealing impact due to its potential properties as a cost-effective alternative to other alloys and its environmental friendliness^34^. Many studies have been dedicated to investigating and improving the physical properties of Zn-Sb alloy^35,36^. After a thorough inspection of published data, it can be asserted that there is no study on the influence of small amounts of antimony for alloying with Zn metal on the anodic dissolution and passivity of Zn in basic electrolytes. Consequently, it is expected that trace alloying of Sb with Zn diminishes the passive layer generated on the zinc anode surface. Therefore, this decrease in the passive layer increases electronic conductivity. For this reason, the performance of the battery is enhanced, and then its long life is increased. Accordingly, the current work efforts on the impact of trace amounts of antimony for alloying with pure zinc on the performance of anodic dissolution and the passive layer produced on zinc in concentrated basic media. Part I study the anodic polarization measurements using the potentiodynamic and potentiostatic methods. Along, the passivated layer produced on the investigated anodes in the examined solution is characterized by utilizing XRD and SEM analysis. Part II concentrates on the anodic polarization behavior utilizing galvanostatic, EIS, and charge–discharge techniques.

## Experimental

### Materials and chemicals

An estimated weight from KOH (Analytical grade) was dissolved in double-distilled water to obtain a concentration of 6 M. Zinc, and antimony metals with a high purity grade (99.99%) were purchased from Johnson Matthey Chemicals Ltd. By using the fusion procedure, the two metals were utilized to fabricate the two alloys of Zn-Sb (0.5% Sb and 1% Sb). The two metals were combined and placed into evacuated closed silica tubes in the proportions indicated. Then, these tubes were fused for 24 h at 750 °C in a muffle furnace. The tubes in the muffle furnace were shaken every 6 h to ensure milt homogeneity. As previously stated, this molten was quenched in cold water^37^. The alloys were produced as rods. The electrodes were fabricated by individually inserting pure zinc and Zn-Sb alloy rods into a Teflon rod and sealing them with epoxy glue. Each fabricated electrode has an exposed surface area of 0.196 cm^2^ in the solution. As in previous work, XRD and SEM have been utilized to evaluate the prepared alloys and define the phases that formed on the alloy surface^38^.

### Electrochemical investigation

All electrochemical tests were carried out with an Ametek VersaSTAT 4 Potentiostat/Galvanostat. The investigations were achieved in an electrochemical pyrex glass cell of a three-electrode system with a volume of 250 ml. The electrochemical cell design was utilized as published elsewhere^39^. The experiments were achieved using disk electrodes (Zn and Zn-Sb). Before being introduced into the electrochemical system, the working anodes were carefully polished with emery paper of size range 1000–1200 µm, washed with purified ethyl alcohol, and then rinsed in flowing doubly-distilled water. The counter and reference electrodes were utilized as a platinum sheet and a saturated calomel (all potentials are indicated). The anodes were sustained at − 2 V versus SCE in the solution of KOH for 5 min to eliminate any unwanted surface materials and air-formed oxide. Then it was detached and thoroughly shaken to remove the bubbles of adsorbed hydrogen.

The curves of anodic polarization have been performed at a potential ramp rate of 1 mV/s from the potential of the open circuit (*E*_corr._) to + 2.5 V (versus SCE). The potential of anodic direction has been set at a specified fixed potential in the potentiostatic test. Then the variance in current density versus time was recorded (current density-time curves). The content and shape of the corrosion products generated on the anodes' surface in a 6 M KOH were investigated at the applied voltage (at the peak and the passive potential). Then, each electrode surface after the experiment was washed with bi-distilled and dried in a desiccator under nitrogen gas (as inert gas) for 30 min. After that, The electrode was transformed immediately from the desiccator to the XRD holder box, and the XRD measurement took 30 min. Copper radiation was utilized with an accelerating voltage of 30 kV and a filament current of 20 mA to analyze the anodically generated film via an X-ray diffractometer with an iron filter. The exterior surface of the passivated film understudy was examined utilizing a scanning electron microscope (SEM) of Joel.

## Results and discussion

### Anodic polarization using the potentiodynamic measurements

#### Influence of trace Sb alloyed with Zn on its anodic behavior

**Figure 1:**
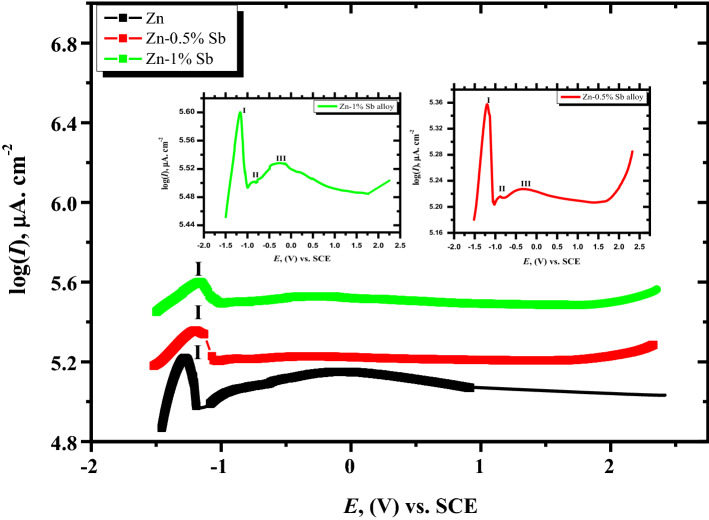
Potentiodynamic polarization behavior in 6 M KOH at 25 °C for pristine Zn metal and its alloys.

The minor antimony alloying with zinc on its anodic behavior in an electrolyte of 6 M KOH utilizing a potentiodynamic technique was performed (Fig. 1). The behavior of potentiodynamic polarization of the pristine zinc and its alloy specimens is initiated from the open-circuit voltage (OCP) to + 2.5 V, with a potential ramp speed of 0.001 V s^−1^ and at ambient temperature (25 °C). The date of the polarization curves exhibited an active/passive behavior. By analyzing the curve of the pristine zinc, one anodic peak in the active dissolution zone is detected, then a passive part comes after. However, the current density at the first part from this passive area (− 1.1 V) starts to slightly raise as shifting the potential to a positive direction up to 0.0 V (SCE), the current density gradually decreases until reaching the steady-state value of the passive region^40^. The anodic peak of pure Zn is observed at around − 1.27 V. It can be attributed to zinc dissolving as Zn^2+^ ions, which combined with OH^−^ ions to generate Zn(OH)_2._ Therefore, the increase in current density to a more positive potential of more than − 1.1 V can be ascribed to the generation and dissolution of zinc hydroxide. On the other hand, the permanently passive area expands throughout an applied potential as the current density decreases till it approaches a tiny value, shifting the potential to more positive values. The observed steady state of current density at more positive potentials can be ascribed to the generation of sufficient Zn(OH)_2_ or ZnO. These findings demonstrate that separating the saturated zincate anions near the surface of the electrode becomes a barrier to zinc dissolution^41^. As a result, the film generated on the exterior Zn surface at the peak potential comprises Zn(OH)_2_ and ZnO. This would be investigated further using SEM and XRD techniques thereafter.

Otherwise, the impact of small amounts of antimony alloyed with zinc is investigated under the same mentioned conditions and technique, as also shown in Fig. 1. The two curves of alloys I and II reveal a similar trend to that observed of pristine zinc. The current of the anodic peak (I) is, nevertheless, greater than the current of the Zn electrode. In addition, the peak of anodic current (I) gradually increases with the increase of antimony alloying with zinc. The findings show that when little Sb is mixed with pristine zinc, the peak voltage (I) moves to higher positive values. Consequently, the obtained data reveal that the impact of a small quantity of Sb with Zn slows down the processes of passivation on the surface of the electrode^42^. Alternatively, the alloys I and II curves exhibit two small peaks with the potential shifting to a more positive direction (II and III) at about − 0.8 and − 0.4 V versus SCE. The small peak potential (II) at − 0.8 V may be ascribed to the dissolution process of the Zn_4_Sb_3_ phase to the Zn-Sb phase and free Sb on the alloy surface. As a result, peak III appears when Sb is oxidized at a greater positive potential (− 0.4 V). El-Sayed et al.^43^ showed that, in a strongly alkaline solution, Sb could be oxidized to the higher state (III) as [Sb (OH)_4_]^−^. Therefore, peak III appeared at about − 0.420 V and is roughly equivalent to the equilibrium potential of Sb_2_O_3_/Sb_2_O_5_.

The permanent passive region can be occurred due to the formation of ZnO and Sb oxides (Sb_2_O_3_ and Sb_2_O_5_). The density of current in the passivated zone of the two alloys, on the other hand, is greater than that of Zn metal, and the current rises as the alloyed Sb quantity rises. This reveals that the passivity diminishes due to adding minor Sb to pure Zn. Consequently, this assists in forming a more conductive layer on the surface of the alloy. Accordingly, the solution easily reaches the surface bulk. This trend exhibits an important role of trace Sb as an alloying metal with zinc metal, which retards the production of oxide on the surface of the alloy^41^. The data obtained from Zn-Sb alloys clear that, with moving the voltage to a more positive potential, particularly at + 2.0 V, the current density of the passivation rises suddenly. That behavior attributes to the oxide film damage generated on the electrode's surface. Consequently, the surface of the alloy becomes active^44^. This behavior can be ascribed to some Sb ions introduced inside the lattice of ZnO^45^. Therefore, minor Sb alloying with Zn has a sensitive influence on passive film breakdown. Consequently, the corrosion processes on the surface become high, resulting in high conductivity. Therefore, the battery recharge can be improved. These results exhibit that small additions of Sb to Zn improve the efficiency to be more suitable for alkaline zinc batteries. This is attributed to the breakdown of the passive layer produced on the surface of the alloy.

#### Temperature Influence on corrosion and passivation

**Figure 2:**
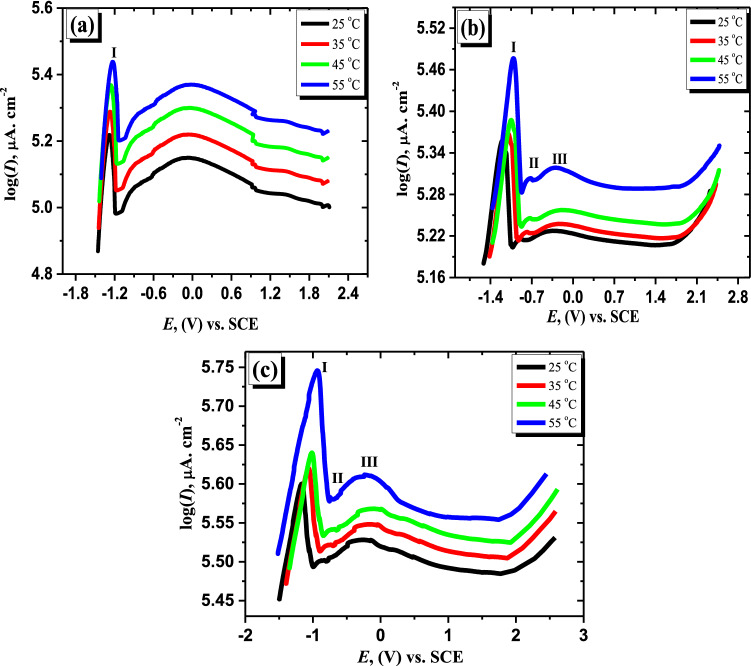
The characteristics of anodic potentiodynamic polarization in the alkaline solution for Zn (**a**), alloy I (**b**), and alloy II (**c**) at various temperatures.

Using the potentiodynamic technique, the anodic polarization attitude of the examined anodes in the mentioned electrolyte at various temperatures (25–55 °C) was studied. The data exhibit that the currents of passivation and peaks of potentials are steadily increasing. While, with the increase in temperature, the corresponding peak potentials move a little bit to the positive value. That trend reveals that the temperature increase influences retarding the oxide film produced on the surfaces of all studied anodes. This manner can be attributed to the generation of an anodic layer that is partially dissolved. Therefore, the rising temperature leads to high migration and diffusion rates for products and reactants^46^. Accordingly, the potentials of peaks are moved to more positive values, and consequently, their currents are increased. For this reason, the passivation is delayed, and the rate of corrosion is increased^47^. The curves of b and c in Fig. 2 of the prepared alloys (I and II) with two different contents of Sb under the same mentioned conditions reveal two small peaks (II and III) in addition to peak I, which is observed in the case of the Zn electrode (Fig. 2a). By inspection of these data, it is shown that the current of peaks of the two studied alloys also increases with elevated temperature, and the potential of peaks is moved to a positive direction. In addition, this trend shows a similar attitude to that observed in the pure zinc electrode. The above findings may be explained by the fact that when the temperature is raised, the diffusion rate of ions rises, and as a consequence, dissolving processes occur^46^.

It's worth noting that the peak current and the current of passivation become higher as the antimony content in the alloy is increased. Besides, the two studied alloys reveal a higher current density of both peaks and passivation compared with that of the Zn electrode at all investigated temperatures. The acquired results lead to the conclusion that the alloy's surface is becoming less resistant to anodic dissolving processes. This is due to that the formed Sb oxides (Sb_2_O_3_ or Sb_2_O_5_) are less stable in concentrated alkaline solution compared with that of ZnO^48^. These findings demonstrate that in a strong alkaline solution, the dissolution of Sb oxides on the alloy surface produces soluble [Sb(OH)_4_]^−^, leading to increasing the density of current^43^. As a result, the polarized Zn atoms and OH^−^ ions are able to interact more easily.

**Figure 3:**
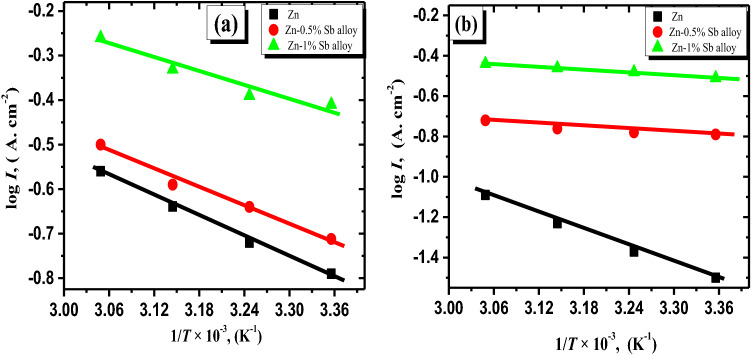
Arrhenius plots for Zn and Zn-Sb samples in the alkaline solution at varied temperatures and fixed potentials of − 1.27 V (**a**) and + 0.8 V (**b**).

From the relevance among log I and 1/T as observed in Fig. 3, the energy of activation for active anodic discharging and passivity processes of the pristine Zn and its alloyed anodes were estimated. The received data show that the values of activation energy (Ea) of pure Zn electrode are higher than these of the two investigated alloys at both peak voltage (I) and passivated zone (Table 1).

**Table 1:** Apparent activation energy values of Zn and Zn-Sb alloys calculated from potentiodynamic anodic polarization curves at different temperatures in 6 M KOH solution.

Metal and alloy	*E*_a_ (kJ mol^−1^) *I*_p_ vs. 1/*T* at peak (A_I_)	*E*_a_ (kJ mol^−1^) *I*_passive_ vs. 1/*T* at passive region
Zn	36.5	86
Zn-0.5%Sb alloy	34.5	72
Zn-1%Sb alloy	28.9	69

This implies that including small amounts of Sb in zinc leads to a lower energy barrier and consequently accelerates the anodic dissolution processes^41^. These results confirm this illustration that in a concentrated alkaline solution, the dissolving process of the examined alloys is faster than the pristine zinc.

### Anodic polarization at different potentials using potentiostatic technique

**Figure 4:**
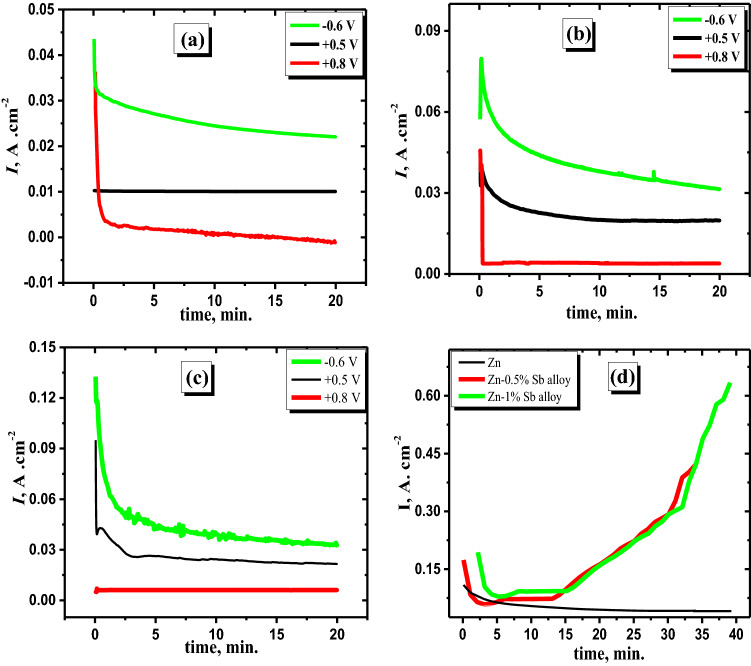
Potentiostatic (current–time) data for Zn (**a**) and its alloys I (**b**) and II (**c**) in 6 M KOH at various potentials as labeled and comparison between Zn and its alloys at breakdown voltage + 2.0 V (**d**).

To confirm that the anodic density of current increases due to the minor Sb alloying with Zn at passive regions, the potentiostatic technique is applied at different anodic potentials (− 600, + 500, and + 800 mV versus SCE). The data in Fig. 4 exhibit the current–time curve for the zinc electrode and its studied alloy specimens in concentrated basic media at 25 °C. It is clear that, in the first few seconds of the applied potential, the current sharply decreased with time until reaching a steady state of the current. As noted in the second stage, the current density seems to decline slightly with time. Those results illustrate that the first step for Zn and its studied alloys regarding steeply decaying of the current can be attributed to the production of Zn(OH)_2_ or ZnO on the surface of the pristine Zn and Sb oxides in addition to ZnO on the surface of the alloy. As the degree of the surface coverage with mentioned oxides increases, the current density slightly diminishes. Furthermore, the steady state of current decreases as the applied potential is shifted to more positive values, which can be assigned to the generation of a thick oxide film. Nevertheless, the current density of the steady-state for the examined alloys is found to be greater than that of pristine zinc. This demonstrates that the inclusion of Sb as an alloying element with Zn causes the Zn-Sb alloy's passivity to deteriorate. This observation may be assigned to the oxidation of Sb to Sb_2_O_3_ or Sb_2_O_5,_ which can be unstable in the concentrated alkaline solution, in addition, to preventing the production of Zn(OH)_2_. However, the production of Sb oxides on the surface of the alloy at higher anodic positive potentials has the aptitude to dissolve in concentrated alkaline solutions yielding soluble [Sb(OH)_4_]^−^ species. As a result, the density of anodic current rises compared with that of pure Zn at the steady state of current density. It's worth noting that the difference in the current density value (at the steady-state region) is lower in the case of pristine zinc in comparison to that of the Zn-Sb samples at the different investigated potentials. This implies that the rate of production for the passivated film on the surface of pristine zinc is higher than that of the surface of the alloy at all examined potentials. Finally, It is possible to deduce that the minor Sb alloying with Zn diminishes the passivation on the surface of alloy electrodes^41^.

The data in Fig. 4d show a comparison between the potentiostatic behaviors for the investigated electrodes in the concentrated alkaline media at an applied potential (the breakdown potential of oxide film at + 2.0 V). It's worth noting that the initial current density of the pristine Zn is lower than that of the alloys. Then, the current jumps suddenly to very high values after a certain time for alloys I and II only but is still stable for Zn through the measurement. This indicates that antimony, the minor alloying element with zinc, is significant in the breakdown of the passive coating on the alloy surface at a specific anodic potential^44^. The presence of Sb on the surface of the alloy can reactivate the surface and, consequently, the conductivity of the Zn^45^. Accordingly, the obtained results are in accordance with the potentiodynamic performance of the examined anodes of the minor Sb alloying with Zn.

**Figure 5:**
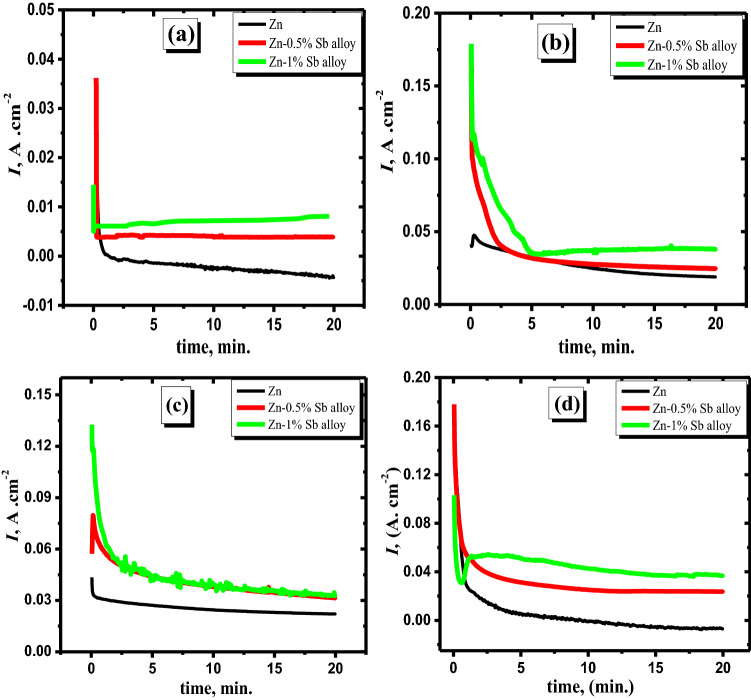
Current–time patterns for zinc and its alloys in 6 M KOH at potentials of − 0.6 (**a**), + 0.5 (**b**), + 0.8 (**c**), and + 1.6 V (**d**) at 25 °C.

Figure 5 shows the measured current densities versus time for the pristine zinc and its investigated alloyed samples at different loading voltages in the passivated area (− 0.6, + 0.5, + 0.8, and 1.6 V). The current densities at the steady-state in the two analyzed alloys are higher than those of pure Zn at all tested potentials, as shown by the results in the cited curves. El-Sayed et al.^40^ showed that the kinetic generation of the anodic passive layer could be revealed as follows:

1 $$I=A {t}^{-n}$$

2 $$\log(I) =\log{A}-n\log{t}$$

where the constant values are *A* and *n* depending on the potential limitations and the solution concentration.

**Figure 6:**
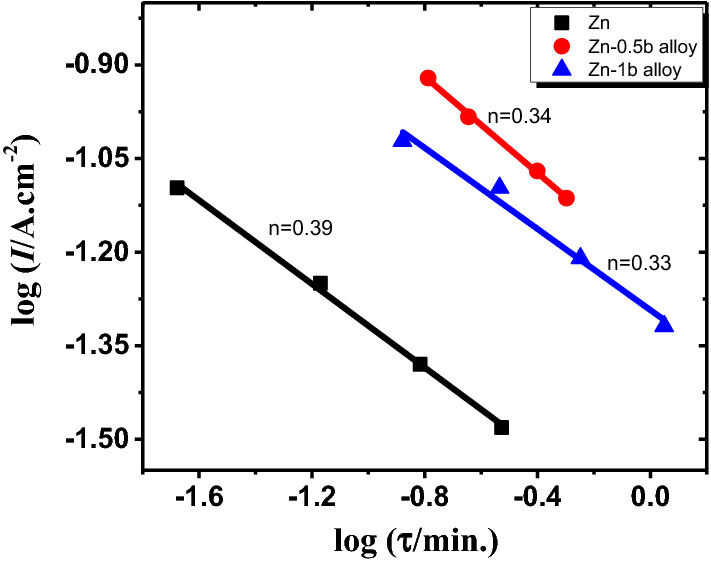
Logarithmic relationship between the duration of the anodic arrest and the density of current for the pristine zinc and its alloys I and II in 6 M KOH at 25 °C.

Additionally, the n value illustrates the oxide formation rate on the surface^49^**.** Therefore, the value of n can be evaluated from the slope of the relation between log *I* and log *t* as a straight line in Fig. 6. The evaluated results reveal that the value of n becomes lower with the addition of Sb to Zn, and it is decreased from 0.39 of pure zinc to 0.33 with Sb alloying with Zn. This suggests that slight Sb alloyed with zinc causes the passivation process to be slowed down on the alloy surface. This illustration is supported by the obtained results of XRD and SEM.

#### Characterization of the passivated layer produced at various applied potentials

**Figure 7:**
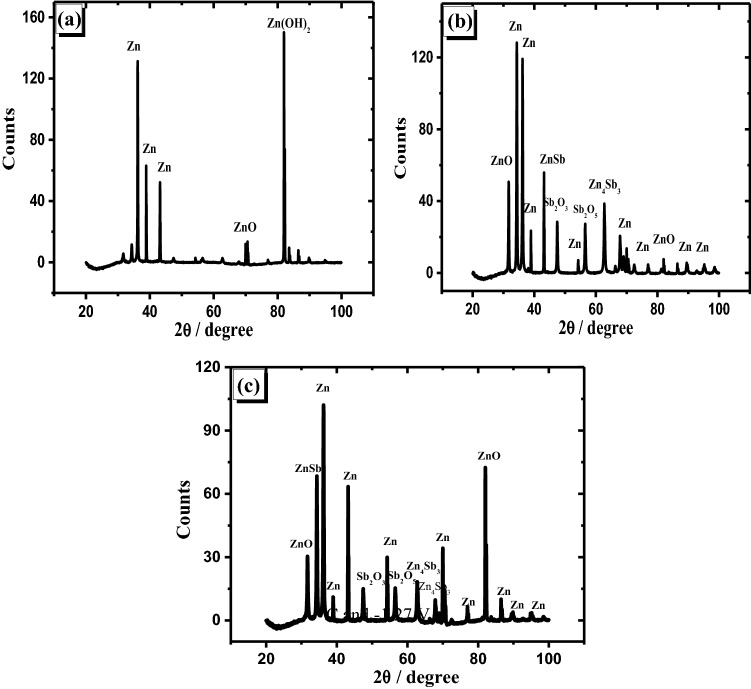
Patterns of XRD for anodically generated oxide film on zinc (**a**) and its alloys I (**b**), and II (**c**) in 6 M KOH at 25 °C and − 1.27 V.

In order to determine the composition of the passive film generated on each studied surface, the potentiostatic technique was used for 20 min in the studied electrolyte at various fixed potentials. Each electrode surface after the experiments was prepared for both XRD and SEM investigations according to the previous work^40^. The XRD patterns of the passive layer formed at − 1.27 V (peak potential) of both zinc and its studied alloy with various antimony content in the mentioned electrolyte have been shown in Fig. 7a–c. Different clear oxides formed on the surfaces are noticed by comparing XRD data (Fig. 7) of pure zinc and its studied alloy electrodes. As referred before in our earlier work^40^, ZnO and Zn(OH)_2,_ in addition to Zn, are identified on pure Zn surface (Fig. 7a). Due to the alloying of Sb with Zn, different oxides are produced on alloy specimens' surfaces such as Sb_2_O_3_ and Sb_2_O_5_ with the presence of ZnO. In addition, ZnSb and Zn_4_Sb_3_ phases with Zn as metal are detected^48^.

**Figure 8:**
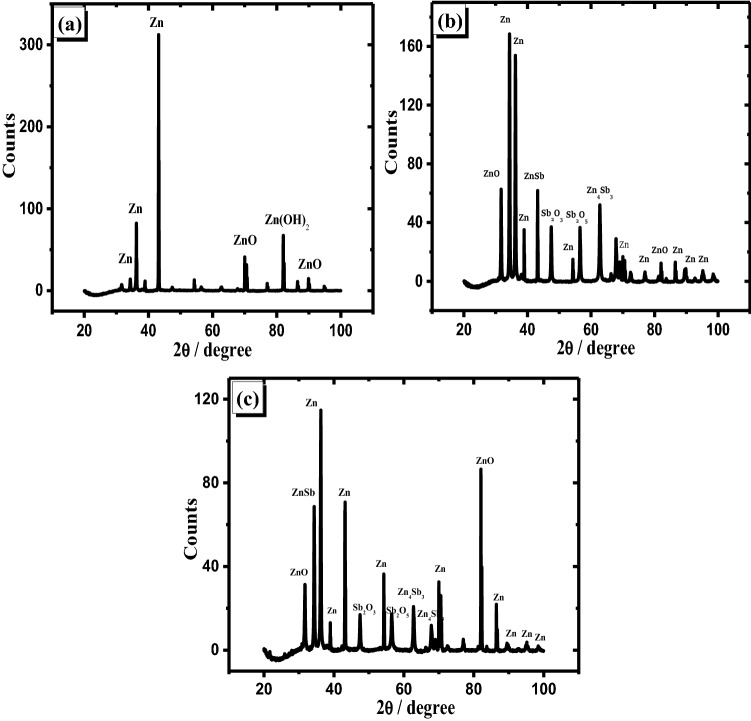
Patterns of XRD for the anodically generated oxide film at + 0.8 V on the pristine zinc (**a**) and its alloys; I (**b**) and II (**c**) in 6 M KOH at 25 °C.

It is interesting to notice that zinc hydroxide vanished due to adding antimony to zinc, and the amount of ZnO increased. Therefore, this addition has the ability to prevent the generation of Zn(OH)_2_ on the surface of the alloy specimen. Also, the formation of Sb_2_O_3_ and Sb_2_O_5_ relatively diminished with the rise of Sb quantity in the alloy. This indicates that part of the amount of Sb oxides dissolves in the examined solution. The analysis of the passivated layer produced on the investigated anodes at + 0.8 V (passive region) using the patterns of XRD has been shown in Fig. 8. It is noticed that the amount of Zn(OH)_2_ which is formed on Zn was decreased compared with that noticed at the potential of the peak.

This shows that when the potential increases, some Zn(OH)_2_ dissolves or loses water molecules and turns to ZnO. Consequently, the current density obtained from potentiodynamic measurements is decreased at + 0.8 V. Figure 8b,c results also reveal that mixed oxides from Sb_2_O_3_ and Sb_2_O_5_ with ZnO have covered the alloy surfaces (I and II). However, the amounts of both Sb_2_O_3_ and Sb_2_O_5_ are lower on the surface of sample II than that of sample I. The pattern exhibits that the higher the Sb level in an alloy, the easier it is to dissolve the oxide layer. As a result, XRD data supports the greater current densities reported in the potentiodynamic and potentiostatic tests of the studied alloy with various Sb contents.

**Figure 9:**
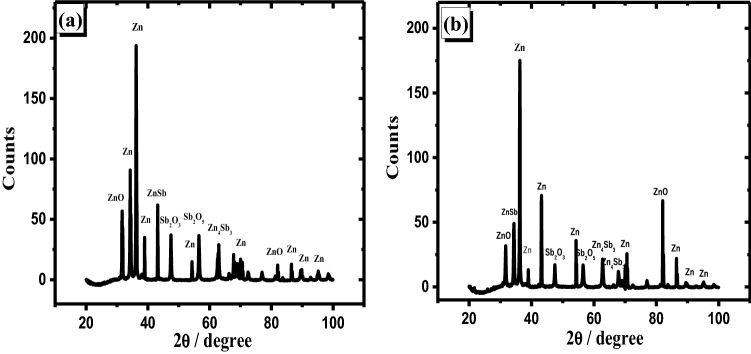
XRD examination of the oxide film generated anodically at the trans-passive potential + 2.0 V on alloy I (**a**) and alloy II (**b**) in 6 M KOH at 25 °C.

XRD spectrum of alloys I and II at the passive layer's breakdown potential (at + 2.0 V) are exhibited in Fig. 9. Although the Sb percentage as an alloying element in specimen II is larger than that of specimen I, the intensities of the peaks of Sb oxides (Sb_2_O_3_ and Sb_2_O_5_) synthesized on the surface of sample II are lower than that of sample I. Alternatively, the ZnSb and Zn_4_Sb_3_ phases on alloy II are smaller than those on alloy I. It also implies that a portion of these phases decomposes faster on alloy II than on alloy I at the aforesaid breakdown potential (+ 2.0 V vs. SCE). As a result of the high Sb percentage in the alloy at a greater positive potential, high desolvation of the oxides in the alkaline media, as well as partial dissociation of the two phases, occurred. Finally, XRD results and interpretations are supported by the sudden increase in the current densities of potentiodynamic and potentiostatic measurements for the examined alloys I and II samples at + 2.0 V (breakdown potential).

#### Examination of the passive film using SEM

**Figure 10:**
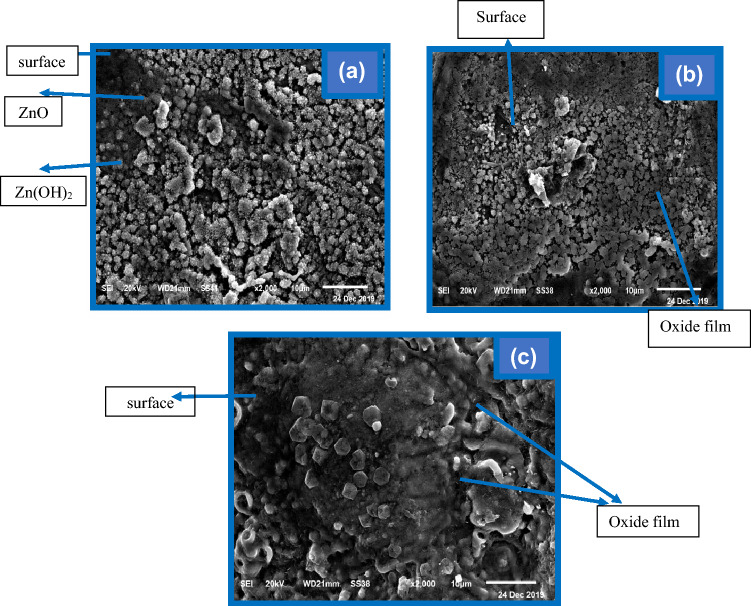
Captured SEM images for the anodically produced film at pristine zinc (**a**), and its alloys I (**b**), and II (**c**) at − 1.27 V in the 6 M KOH solution.

The data in Fig. 10 exhibit the photographs of the anodically passivated layer produced utilizing the potentiostatic technique in the investigated electrolyte at the potential of the peak (− 1.27 V) of the investigated anodes. The passivated film generated on the pure Zn surface at the mentioned peak is shown in Fig. 10a. The surface appears to be totally covered by a thick layer containing various particle morphologies that might be correlated to a Zn(OH)_2_ and ZnO combination. Nevertheless, applying 0.5 percent Sb to Zn reduces the amount of oxides on the surface, resulting in certain sites seeming to be uncovered by oxides (Fig. 10b). Furthermore, there is a crossover between the various Zn and Sb oxides. Figure 10 clearly shows an SEM image of the alloy II surface (1% Sb). It is noticed that small quantities of oxides are produced, so some parts from the surface can be observed. It is also noticed that small quantities from the oxides are generated, and larger parts from the surface are bared compared to those of the Zn-0.5%Sb sample. This illustrates that the oxidation of the unalloyed Sb to its oxides on the surface of the alloy at the mentioned peak is low.

**Figure 11:**
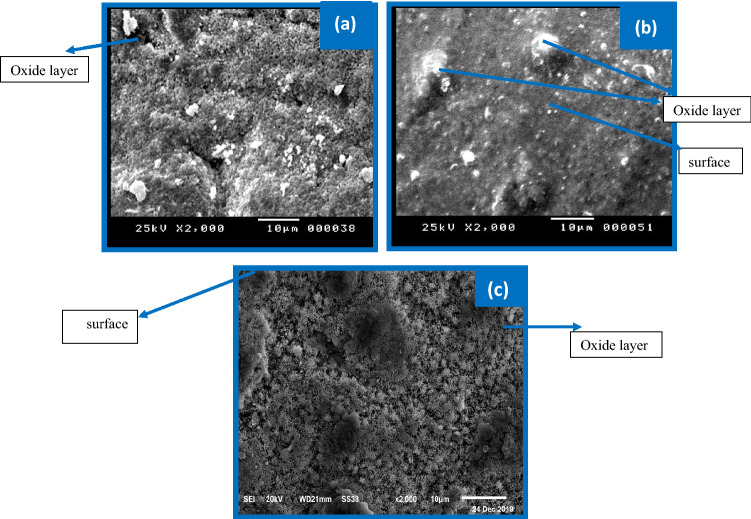
Captured images of SEM for the generated film of pristine zinc Zn (**a**), and its alloys I (**b**), and II (**c**) at + 0.8 V in the 6 M KOH electrolyte.

Figure 11a–c illustrates the captured images by SEM of the anodically passivated film produced at + 0.8 V on the pristine Zn and its alloys I and II, respectively. In general, the results revealed that the investigated anodes' surfaces are coated by some more small particles than those at the peak potential. Notably, the Zn surface appears to be entirely covered by the oxide layer. However, the layer formed on the surface of the alloy decreases with the increase of Sb quantity so that some bare parts from the surface can be seen (Fig. 11c). This proves that the dissolution of the oxides produced on the surface of the alloy is higher than its formation.

**Figure 12:**
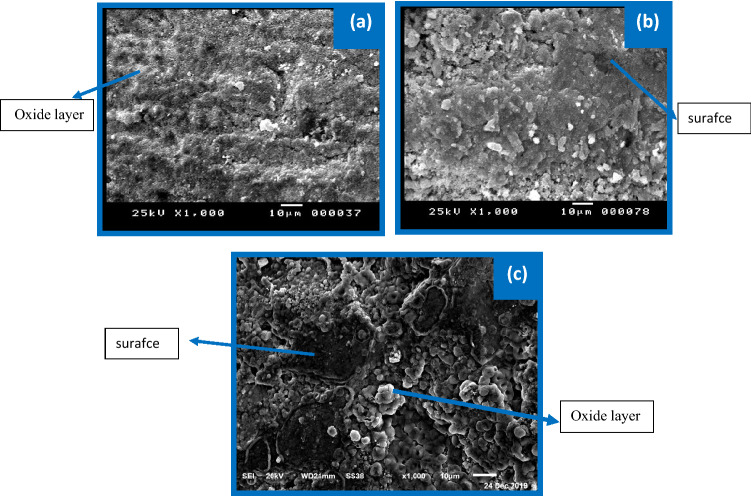
Captured images of SEM for the produced layer of pristine zinc (**a**), and its alloys I (**b**), and II (**c**) at + 2.0 V in the 6 M KOH electrolyte.

It can be seen from Fig. 12a that the layer formed seems to be thick and adhered to the Zn surface at + 2.0 V (vs. SCE) compared with that at a lower positive voltage (+ 0.8 V). This phenomenon showed that the surface of Zn becomes more protective as the voltage increases to a more positive value. However, the data of SEM of alloys I and II exhibited opposite behavior; that is, a few amounts from oxide particles are observed on the surface (Fig. 12b,c) compared with those on the Zn surface. Besides, some parts from the surface seem to be uncovered by oxides film, particularly for alloy II (1%Sb). This finding may be due to the alloy's Sb presence, which causes the passive layer to break down. This phenomenon explains why the current density of potentiodynamic and potentiostatic measurements at + 2.0 V (SCE) in the tested alloys was unexpectedly elevated. Data suggests that alloying Sb with Zn aids in dissolving the passive layer in concentrated alkaline conditions^40^. In more detail, the rupture of the passivated layer at a certain voltage related to the alloying of Sb with Zn is due to the presence of Sb ions in the ZnO film. As a result, the passivated coating on the alloy surface is more vulnerable to rupture.

## Conclusions

The present work studies the effect of adding minor Sb with various contents to Zn on the performance of anodic dissolution and passivity of zinc in concentrated basic media. Two different techniques were used in the measurements, such as potentiostatic and potentiodynamic. The results exhibited that the currents of anodic dissolution in the active region and passivation are higher with minor Sb alloying than those of Zn. This illustrates that including a small quantity of antimony in zinc facilitates the anodic dissolution in the active zone and retardation of the passive layer production at higher positive voltage. The rising temperature has significantly influenced an increase in the density of current for both passive and active zones of all studied anodes. The energy values of activation (*E*_a_) in the two mentioned regions diminish with increasing the included Sb to Zn. Therefore, the highest dissolution and lowest passivation rates were observed at 1%Sb. It means that using little Sb as an alloying metal with Zn helps to make the layer generated on the alloy's surface more conducive. Consequently, the solution easily reaches the surface bulk. The data showed that at a more positive voltage (+ 2.0 V), the current density of passivation sharply increases. This suggests that the oxide layer produced on the surface is demolished. Consequently, its surface becomes active. Therefore, the non-conducting layer generated on the surface of Zn can be hindered by adding Sb to Zn. Accordingly, the battery recharging can be improved. The measurements using the potentiodynamic technique exhibited that the inclination of the examined electrodes towards passivity increases with the shifting of potential to more positive values. The data showed that the current densities at the steady state of the two examined alloys were greater than those of pristine zinc at all selected potentials. The oxide growth rate (n) on the alloy surface is lower than that of Zn. This result proves that Sb alloying with Zn retards the passivation process. The revealed data utilizing potentiostatic measurements confirm the potentiodynamic performance of the studied electrodes. The analysis of XRD and SEM showed that the passive film formed on Zn contains ZnO and Zn(OH)_2_, and the amount of Zn(OH)_2_ decreases with moving the voltage to higher positive values. Nevertheless, the surface of Zn-Sb samples includes Sb_2_O_3_ and Sb_2_O_5_ in addition to ZnO. This indicates that Zn(OH)_2_ production is prohibited by including small amounts from Sb to Zn.

## Data Availability

All data generated or analysed during this study are included in this article.
